# Glycolytic reprogramming during microglial polarization in neurological diseases

**DOI:** 10.3389/fimmu.2025.1648887

**Published:** 2025-10-07

**Authors:** Xiaoting Li, Congcong Fang, Yina Li, Xiaoxing Xiong, Xu Xu, Lijuan Gu

**Affiliations:** ^1^ Department of Anesthesiology, Renmin Hospital of Wuhan University, Wuhan, China; ^2^ Central Laboratory, Renmin Hospital of Wuhan University, Wuhan, China; ^3^ Department of Neurosurgery, Renmin Hospital of Wuhan University, Wuhan, China; ^4^ Department of Geriatrics, Renmin Hospital of Wuhan University, Wuhan, China

**Keywords:** microglia, glycolysis, lactylation, Warburg effect, metabolic reprogramming, nervous system diseases

## Abstract

**Background:**

Microglia, the resident immune cells of the central nervous system (CNS), play pivotal roles in the onset and progression of various neurological disorders. Owing to their remarkable plasticity, microglia can adopt diverse phenotypic states in response to distinct microenvironmental cues. Over the past decades, accumulating evidence has demonstrated that immune cell metabolism critically regulates their polarization and effector functions through a process termed metabolic reprogramming, in which glucose metabolism is particularly central. Glycolytic reprogramming underlies the entire polarization process, and elucidating its mechanisms may enable targeted modulation of microglial activity to mitigate their deleterious effects in CNS pathologies, thereby offering novel therapeutic avenues for these diseases.

**Aim of the Review:**

This paper summarizes what is known about microglial polarization and glycolytic reprogramming and explores their important roles in the development of neurological diseases. The link between microglial metabolomics and epigenetics in neurological disorders requires further study.

**Key Scientific Concepts of the Review:**

Microglia exhibit distinct phenotypic states at different stages of central nervous system (CNS) disorders, and these polarization processes are closely coupled with glucose metabolic reprogramming. Proinflammatory microglia predominantly rely on glycolysis, whereas reparative or anti-inflammatory phenotypes primarily utilize oxidative phosphorylation. Targeting glycolytic pathways to limit the polarization of microglia toward proinflammatory states has emerged as a promising therapeutic strategy for CNS diseases.

## 1 Introduction

Microglia are common resident immune cells in the central nervous system (CNS). Their primary functions include immune surveillance, immune defense, phagocytosis, and nutritional support; thus, their roles in maintaining homeostasis and repairing tissue in the CNS cannot be ignored (1, 2). Inflammation is part of a highly conserved endogenous response to organ injury or disease (3–5). Thus, microglia are involved in the response to almost all types of neurodegeneration, stroke, and brain tumors.

In the past, microglia were considered “resting” cells and were activated only in response to stimuli such as infection or injury. However, with further research, the surveillance function of microglia in the CNS has gradually been recognized. When confronted with stimuli such as foreign pathogens, abnormally aggregated proteins, and apoptotic cells, microglia detect homeostatic changes in the brain environment through a highly motorized process of retraction and extension (4). They also undergo chemotaxis and perform phagocytosis by continuously altering the cytoskeleton; removing cellular debris and apoptotic neurons; sensing subtle changes in the microenvironment, such as changes in immunoglobulin and adhesion molecule levels and the presence of inflammatory stimuli (6); performing immune surveillance and maintaining microenvironmental homeostasis (4, 7–9); and transitioning from a highly branched resting state to an amoeboid state (10, 11). Thus, microglia are often the primary activated neuroglia. However, while the activation of microglia can have a positive effect, it can also contribute to hindering nervous system repair and exacerbating tissue damage. Microglia have contradictory functions in different stages of nervous system injury (12, 13).

In fact, microglia exhibit heterogeneity and are capable of adaptively modulating their functions in response to environmental changes (14).

Immune cells can regulate intracellular metabolic processes to modulate the initiation, intensity, and duration of immune responses (15). As the resident immune cells of the central nervous system, microglia also undergo metabolic alterations upon activation. This review focuses on alterations in glucose metabolism, specifically glucose metabolic reprogramming. It summarizes current advances in understanding glucose metabolic reprogramming in activated microglia and discusses its association with representative central nervous system diseases.

## 2 Neuroinflammation and microglial polarization

The inflammatory response is an important innate immune response to neurological disorders. Microglia constitute the first line of defense in the innate immune response, and overactivated microglia contribute to the progression of neurological diseases by releasing various proinflammatory factors to create an inflammatory microenvironment.

**Figure 1:**
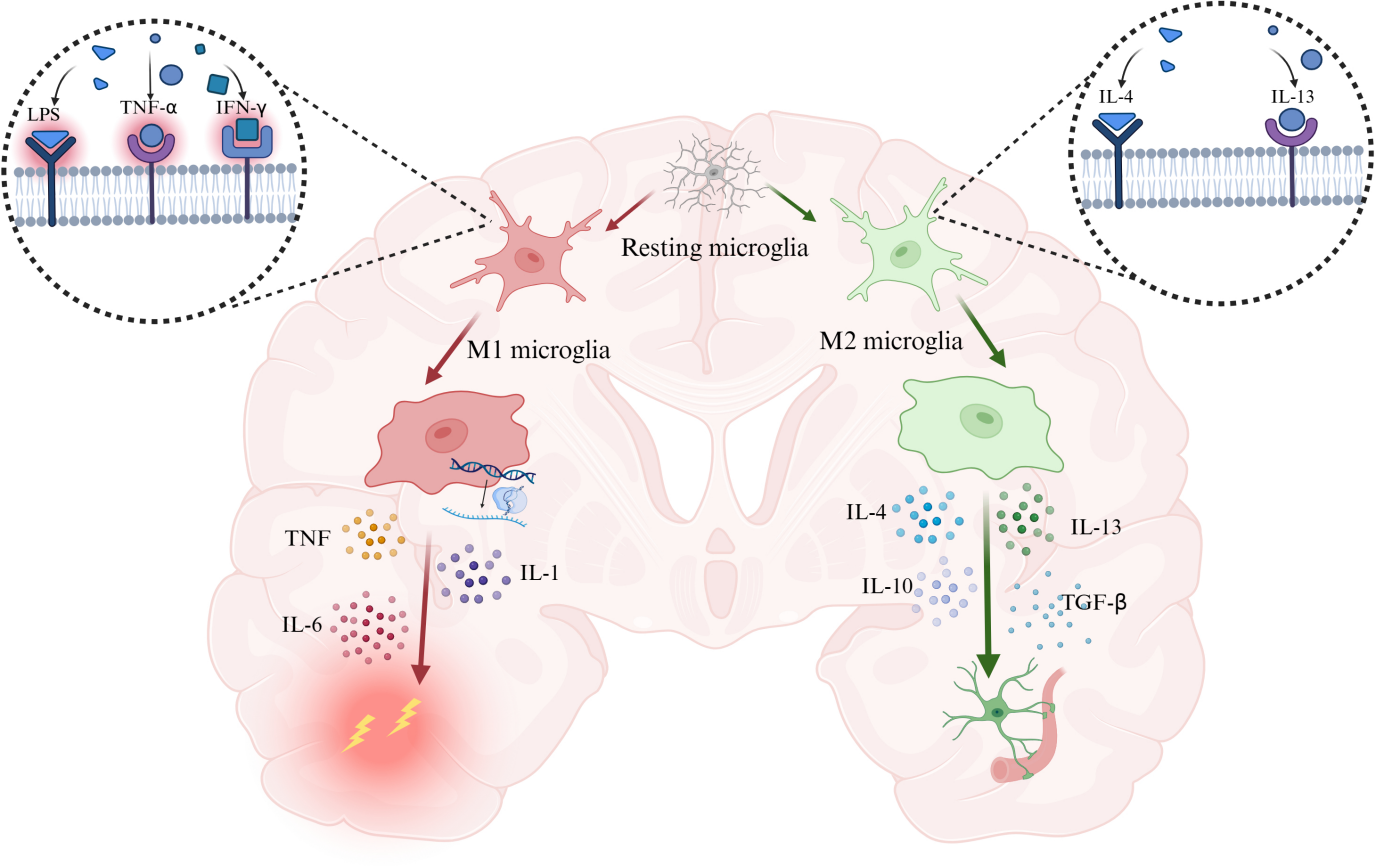
PAMPs and DAMPs drive microglia toward a pro-inflammatory, injury-promoting phenotype characterized by the secretion of TNF, IL-6, and IL-1, whereas IL-4 and IL-13 induce a reparative phenotype that releases IL-4, IL-13, IL-10, and TGF-β to suppress inflammation and promote tissue repair. Abbreviation: IL-1, interleukin-1; IL-4, interleukin-4; IL-6, interleukin-6; IL-10, interleukin-10; IL-13, interleukin-13; IFN-γ, interferon-γ. Created with BioRender.com.

Under normal circumstances, pattern recognition receptors (PRRs) initiate the host inflammatory response. By recognizing pathogen-associated molecular patterns (PAMPs) and damage-associated molecular patterns (DAMPs), PRRs enhance the transcription of inflammation-related genes and promote the release of pro-inflammatory cytokines, type I interferons, and chemokines (16). Among them, tumor necrosis factor (TNF), interleukin-6 (IL-6), and interleukin-1 (IL-1) play central roles in the inflammatory response. They regulate cell death, alter vascular endothelial permeability, recruit immune cells to amplify inflammation, and induce the production of acute-phase proteins (17). Microglia can sense inflammatory signals initiated within the central nervous system, leading to alterations in their activation state (18, 19), and actively respond by transmitting signals. Microglia can act on astrocytes to induce further immune responses through the secretion of inflammatory factors such as TNF-α and IL-1β (20). Thus, microglia play an important role in inflammatory responses in neurodegenerative diseases (21) (**Figure 1**).

Research on microglia has been ongoing for over a century. Before the 21st century, researchers’ understanding of microglia was largely limited to their resting state under physiological conditions and their abruptly altered activated state in pathological environments. In 2005, using *in vivo* two-photon imaging, researchers discovered that microglia in the so-called resting state are not truly “static.” Even under physiological conditions, they exhibit highly dynamic synaptic activity, serving a housekeeping role that maintains cerebral microenvironmental homeostasis and enables rapid responses to brain injury (4). This finding corrected earlier assumptions, revealing that microglia do not become activated only in response to pathological stimuli, but rather remain continuously active, enabling timely adaptation to diverse environmental challenges (22). Initially, drawing on the classification of macrophages, immunologists adopted a simplified dichotomy for microglia, categorizing them into “M1” classical activation associated with pro-inflammatory and neurotoxic effects, and “M2” alternative activation associated with anti-inflammatory and neuroprotective functions. Subsequently, as research advanced, the dichotomous concept was increasingly questioned, since the polarization process is inherently dynamic and functional overlap exists among different phenotypes. Single-cell sequencing and transcriptomic analyses have revealed that, in the *in vivo* environment, microglia commonly co-express both M1 and M2 markers (23–25). Acutely activated microglia exhibit heterogeneity (26), with transcriptomic profiles that differ markedly from those of microglia in chronic neurodegenerative conditions (14). In fact, microglia dynamically modulate multiple signaling pathways in response to environmental cues, and their exceptionally sensitive adaptability gives rise to complex cellular states and functions, making the dichotomous classification inadequate for accurate description. With technological advances, single-cell approaches, multi-omics, and analyses of gene and protein expression have provided new insights into the classification of microglia. Their unique heterogeneity enables microglia to effectively adapt to changes in the cerebral microenvironment, exhibiting diverse functions and undergoing morphological alterations. New classifications of microglia are gradually being reported. In 2023, Ma et al. classified eight subpopulations of microglia by single-cell sequencing in a mouse model of ischemic stroke and reported that gene expression was continuous across the different classifications, which supports the continuity of the process of microglial differentiation (27). New categories of microglia have been continuously proposed, such as disease-associated microglia (DAM) related to Alzheimer’s disease (28), microglia of neurodegenerative disease (MGnD) (29), multiple sclerosis–associated inflammatory microglia (MIMS) (30), and lipid droplet–accumulating microglia (LDAM) identified in aging models (31). The functions of microglia in different states are not merely pro-inflammatory or anti-inflammatory but rather display distinct characteristics. Therefore, the classification and nomenclature of microglia across various states should adopt a more refined and multidimensional framework (22).

## 3 Glycolytic reprogramming during microglial polarization

### 3.1 The Warburg effect (glycolytic reprogramming) in microglia

**Figure 2:**
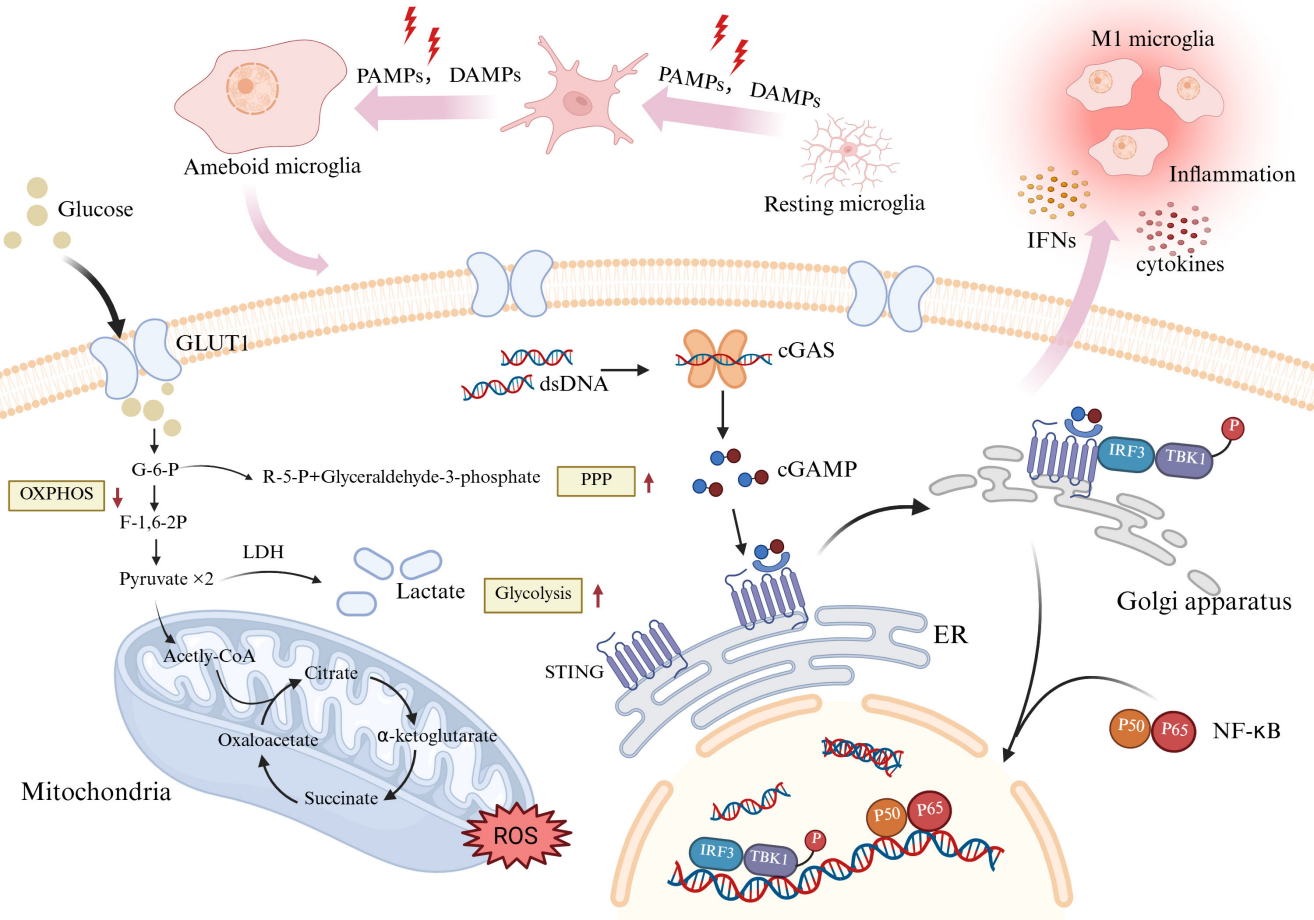
Activated microglia accumulate around damaged brain tissue and phagocytose Aβ and neurofibrillary tangles. Meanwhile, Aβ protofibrils stimulate relevant proteins on the surface of microglia, activate specific pathways to promote the secretion of inflammatory factors, thereby exacerbating the pathological changes of AD. Abbreviation: Aβ, amyloid β; CD36, cluster of differentiation36; CD47, cluster of differentiation47; NLRP3, NOD-like receptor thermal protein domain associated protein 3. Created with BioRender.com.

The central nervous system has unique metabolic energy requirements. The brain consumes 20% of the body’s glucose and oxygen, even though it accounts for only 2% of the body’s weight (32). The energy needs of the brain depend on the supply provided by the peripheral circulation. Research on energy metabolism in the brain has focused mainly on astrocytes and neurons. Microglia, the ‘immune guardians’ of the brain, comprise only 10–15% of brain cells, and their energy metabolism has been poorly studied (33–36). Microglia are responsive to external stimuli. Especially at the level of sugar metabolism, a gene expression analysis revealed that microglia express genes that are essential for glycolysis and OXPHOS (37). Glycolysis involves the metabolism of glucose to pyruvate, which enters the tricarboxylic acid cycle and usually undergoes oxidative phosphorylation. When stimulated by inflammation, the metabolic pathway of microglia changes, and pyruvate metabolism tends to favor glycolysis to produce lactic acid, even in an oxygen-rich environment. This phenomenon is analogous to the Warburg effect in tumor cells, and with reference to this concept, the field of immunometabolism has introduced a new concept that is relevant to tumors: the activation and function of immune cells can be controlled by the regulation of cellular metabolism, termed metabolic regulation or metabolic reprogramming (**Figure 2**). In 2020, Hu et al. reported that microglia can switch from oxidative phosphorylation to glycolysis in response to different stimuli (38). Glycolysis becomes the dominant energy metabolism pathway in microglia in response to injurious stimuli. Although glycolysis is capable of producing less ATP than mitochondrial oxidative phosphorylation, it metabolizes glucose 10–100 times faster than OXPHOS, enabling the cell to satisfy the significant energy requirements of energy-demanding activities such as migration, proliferation, phagocytosis, and cytokine secretion (39, 40). Cellular experiments have shown that cell metabolism also shifts toward the pentose phosphate pathway (PPP) in activated BV-2 microglia (41). Microglia can activate the PPP in parallel with glycolytic reprogramming. The PPP is a metabolic pathway that accompanies glycolysis. Nicotinamide adenine dinucleotide phosphate (NADPH), produced by the PPP, is a reducing agent in anabolic reactions and can contribute to nitric oxide (NO) production. NO and HIF-1α inhibit pyruvate dehydrogenase and indirectly inhibit the tricarboxylic acid cycle, forcing cells to use glycolysis exclusively as a source of energy (42).

Upon this metabolic shift, microglia can rapidly produce large amounts of ATP and glycolytic products. Lactic acid metabolized by cellular glycolysis has long been considered a byproduct of energy metabolism, and its function has not received sufficient attention. In fact, lactate can also serve as an important substrate for energy production (43). Intercellular lactate transport depends on monocarboxylate transporters (MCTs), while lactate oxidation relies on lactate dehydrogenase (LDH). Studies have shown that microglia express MCTs (44, 45), and LDHb is among the most highly expressed genes in these cells (46, 47). This indicates that microglia can utilize lactate metabolism through the uptake of environmental lactate (47). With further research, lactate has been found to regulate inflammation-related genes, thereby influencing the activation profile and functional state of microglia. Several *in vitro* studies have shown that direct stimulation with lactate can induce microglia to release pro-inflammatory cytokines such as TNF-α, IL-6, and IL-1β. In contrast, blocking these pro-inflammatory cytokines does not lead to morphological changes associated with microglial activation (48). This suggests that lactate can directly modulate the phenotype and function of microglia. In 2019, *in vivo* experiments demonstrated that lactate can regulate the temporal dynamics of inflammation through histone lactylation, promoting the expression of pro-inflammatory genes in the early phase while shifting toward the activation of anti-inflammatory or reparative genes in the later phase, thereby exhibiting a transition from a “pro-inflammatory” to a “repair” phenotype (44, 46). This process has been termed “lactylation”. Lactylation has revealed a novel pathway of post-translational protein modification. In 2023, Pan et al. reported that histone lactylation at H4K12la can initiate gene transcription by binding to the promoters of glycolytic genes, thereby enhancing the expression of HIF-1α, PKM, LDHA, and other glycolysis-related genes. In the context of Alzheimer’s disease, microglia undergo a metabolic shift toward glycolysis, accompanied by increased histone lactylation, which further promotes glycolysis and establishes a positive feedback loop between metabolism and epigenetic modification (49). According to the study by Wei et al. (2023), hippocampal microglia in aging models exhibit elevated lactate levels, leading to increased H3K18la. The H3K18la/NF-κB signaling pathway mediates inflammation during the metabolic shift from glucose metabolism to aerobic glycolysis, thereby exacerbating brain aging and disease progression in patients with Alzheimer’s disease (AD) (50). Han et al. reported that lactic acid stimulates microglia with a proinflammatory phenotype to shift to a reparative phenotype, which may reduce neuroinflammation, perhaps representing a new target to improve cognitive function and reverse AD (51). In summary, elevated intracellular lactate levels serve as the key driving force of lactylation. Increased lactate acts as a substrate that provides the material basis for histone lactylation, enabling specific histone modifications that target gene promoters and regulate gene expression epigenetically. Through this mechanism, lactate influences microglial function and thereby contributes to disease progression.

Research on microglial metabolic reprogramming is no longer confined to inflammation itself, but has shifted from focusing solely on the state and functions of microglia to examining their effects on other cells. Studies have shown that the loss of Bach1, a key regulator of glycolysis, during microglial development suppresses the expression of critical glycolytic enzymes such as HK2 and GAPDH, thereby reducing lactate production. This results in decreased H4K12la modification and diminished enrichment at the Lrrc15 promoter. Consequently, reduced secretion of microglial Lrrc15 attenuates activation of the JAK/STAT3 pathway, leading to impaired astrocyte generation and a sustained decline in both astrocyte numbers and neurogenesis (52). Studies have shown that knockout of MCT4 impairs microglial synaptic pruning, lactate uptake and metabolism, and lysosomal acidification. These defects result in marked alterations in neuronal synapse number and size, along with a significant increase in the amplitude of spontaneous postsynaptic currents, ultimately leading to neuronal hyperexcitability (47). In 2023, researchers showed that in the context of multiple inflammatory stimuli, only primary microglia exhibit an early burst of glycolysis and produce NO that interferes with oligodendrocyte metabolism, resulting in a shift of oligodendrocytes to glycolysis to maintain ATP levels and ensure oligodendrocyte survival, which in turn alters the maturation of oligodendrocytes to myelinating oligodendrocytes, thus affecting myelin sheath regeneration (53).

Lactate in the brain is primarily produced by astrocytic glycolysis and transported via MCT1 and MCT4 into the astrocyte–neuron interface, where it enters neurons through MCT2. Within neurons, lactate is utilized through the OXPHOS pathway to generate ATP, a process known as the astrocyte–neuron lactate shuttle hypothesis (54, 55). Some scholars have proposed that, under pro-inflammatory conditions, microglia may also shuttle lactate to neurons (46). In 2024, new research revealed that lactate mediates crosstalk between microglia and neurons. Under neuroinflammatory conditions, lactate produced by microglia can be transferred to neurons via the microglia–neuron axis, leading to lipid droplet accumulation, disruption of neuronal metabolism, and increased ferroptosis in neurons (43). However, research on lactate shuttling between microglia and astrocytes, as well as between microglia and neurons, remains insufficiently explored.

### 3.2 Microglial polarization and glycolysis

#### 3.2.1 Microglial polarization, inflammation, and glycolysis

Inflammation is inextricably linked to microglial polarization, and as unique immune cells, polarization is an important hallmark of CNS inflammation. Glycolytic reprogramming is linked with microglial polarization and is an important marker of the proinflammatory activation of microglia (56). A shift in metabolism occurs to meet the energy requirements of microglia after polarization. In the presence of inflammatory stimuli, activated microglia require far more energy than resting microglia to undergo a range of processes, such as deformation, movement, phagocytosis, and secretion. Glycolysis can be used to meet this specific energy need because it is very rapid. Microglial polarization in response to neuroinflammatory stimuli is inextricably linked to the reprogramming of cellular glucose metabolism. As early as 2013, LPS-induced microglial activation was reported to significantly alter metabolism, inhibit mitochondrial function, and increase glycolysis (57).

#### 3.2.2 Mechanisms of the interaction between microglial polarization and glycolysis

##### 3.2.2.1 Glucose transporter proteins

The mechanisms underlying the interplay between inflammation and glycolytic reprogramming due to microglial cell polarization are still under investigation. Glucose from the peripheral circulation passes through the blood-brain barrier (BBB) and enters the central nervous system, where glucose transporter proteins (GLUTs) are channels that play a key role. Microglia express a variety of GLUTs, including GLUT1, 3, 4, 5, 6, 8, 9, 10, 12 and 13 (58). Glucose uptake by microglia depends mainly on GLUT1, and the inhibition of GLUT1 expression reduces glucose uptake by microglia and decreases the production of inflammatory factors. GLUT1 expression is significantly increased after stimulation with LPS and IFN-γ, which further promotes glycolysis. Thus, modulating GLUT1 expression induces the reprogramming of metabolic pathways and inhibits microglial activation to alleviate inflammation and slow neurodegeneration (59, 60).

##### 3.2.2.2 Hexokinase 2

Hexokinase 2 (HK2) is the rate-limiting enzyme that catalyzes the first step of glucose phosphorylation. RNA sequencing data analysis revealed that HK2 is preferentially enriched and specifically expressed in microglia. *In vitro* experiments demonstrated that HK2-deficient microglia exhibit significantly reduced levels of lactate and ATP, indicating that HK2 is a key regulator of glycolysis in microglia (61). The loss of HK2 does not affect microglial homeostasis under normal conditions; however, its deficiency profoundly impairs microglial proliferation and maturation (61). Previous studies have suggested that the inhibition of HK2 suppresses the inflammatory response induced by microglial polarization (62). However, a 2023 study revealed that in ischemic stroke models, microglial HK2 exhibits a dual role. On the one hand, loss of HK2 reduces glycolysis in microglia and delays their regenerative capacity, thereby suppressing inflammation. On the other hand, in response to cerebral ischemia and hypoxia, HK2 deficiency alters mitochondrial membrane potential and increases mtROS, which act as pro-inflammatory activation signals, ultimately exacerbating brain injury and behavioral deficits (61). A 2024 study on hemorrhagic stroke found that, in the early stages of the disease, HK2 expression in microglia is downregulated and glycolysis is impaired, yet this is associated with an enhanced inflammatory response. A similar mechanism has been attributed to mitochondrial dysfunction: inhibition of HK2 increases mitochondrial permeability and decreases membrane potential, leading to enhanced cytochrome c release, which in turn promotes mtROS accumulation and exacerbates disease-related damage (63).

##### 3.2.2.3 Monocarboxylic acid transporter

In 2021, Cheng et al. reported that inhibition of glycolysis suppresses the expression of proinflammatory genes in microglia at the transcriptional level (56). Monocarboxylic acid transporter (MCT) proteins are important transporter molecules that transport lactic acid to the extracellular space. In 2019, Kong et al. reported that knockdown of MCT1 significantly reduced the expression of iNOS, IL-1β, IL-6, and STAT1, thereby inhibiting classical microglial polarization (44). Arg1 regulates NO production, thereby attenuating intracellular damage from oxidative stress, and it is considered a marker of reparative microglia, acting as a reparative agent. A lactate-induced increase in Arg1 expression promotes reparative microglial polarization and accelerates tissue injury recovery (46, 64).

##### 3.2.2.4 Mitochondria

Mitochondria are the energy factories of the cell, are responsible for energy production and a wide range of biosynthetic processes, and are a major source of intracellular ROS. Mitochondria are dynamically active organelles that are constantly moving to meet the energy needs of the cell through fission and fusion. When a cell is exposed to harmful stimuli, mitochondria undergo fission and are degraded in the lysosome, which is known as mitochondrial autophagy (65). In general, mitochondria can make full use of nutrients to produce ATP while meeting the needs of growth and division, resulting in the formation of cellular structures. When mitochondria are damaged, harmful substances accumulate in the cell, but the methods by which to the cell ensures a balance between energy production and biosynthesis are unclear. Recently, new research has revealed that the functional transformation of mitochondria is a dynamic process. When confronted with damaging environmental stresses, mitochondria are divided into two subgroups by the actions of pyrroline-5-carboxylate synthase: one responsible only for the production of ATP and the other responsible for the production of amino acids and the synthesis of new cellular structures. These dual functions allow cells to survive and grow even in injurious environments (66, 67). Alterations in mitochondrial function and metabolism have important implications for microglial polarization and function. Inflammatory stimuli allow microglia to shift the direction of electron transport in mitochondrial complex I, thereby altering its function from the generation of ATP to the generation of mtROS that results in the sustained activation of microglia to mediate neuroinflammation (68). Thus, mitochondria are important organelles that influence microglial activation and function, and mitochondria with different divisions of labor might contribute to the activation of microglia in different directions and with different functions. Mdivi-1 is a mitochondrial fission inhibitor. Mdivi-1 prevents excessive LPS-stimulated microglial fission and inhibits glycolytic reprogramming, reducing the release of cytokines and thereby reducing the inflammatory response (69). NLRP3 is critical for microglial activation. Accumulating evidence indicates that mitochondrial damage can activate the NLRP3 inflammasome via ROS (70, 71). ROS production and activation of the NLRP3 inflammasome are the most important features of injury-associated microglia (72). NLRP3 is also a regulator of metabolism. The NLRP3 inflammasome/IL-1β/PFKFB3 axis is a key pathway influencing glycolysis in macrophages, and inhibition of the NLRP3 inflammasome suppresses glycolysis and the expression of the glycolysis regulator fructose-2,6-bisphosphatase 3 (PFKFB3). This process is mediated by IL-1β. Activating the NLRP3 inflammasome is essential for linking immunity to inflammation and metabolism (73). The blockade of pyruvate kinase repair-associated microglia in the glycolytic pathway limits NLRP3 activation in mouse macrophages (74). The role it plays in microglia is not well researched.

##### 3.2.2.5 PI3k/(Akt/mTOR/HIF-1α)

During the reprogramming of glycolysis, the phosphatidylinositol-3-kinase (PI3k)/protein kinase B (Akt)/mTOR signaling pathway has been implicated in a variety of cellular activities, including inflammation, autophagy and aberrant cell proliferation. PI3k can promote HIF-1α protein translation via Akt and downstream mTOR activation (75) and upregulate the expression of GLUT and glycolytic enzymes, increasing the rate of glycolysis. Inhibition of mTOR activity promotes microglial repair-associated polarization, reduces inflammatory factor production, and inhibits glycolysis (38, 76). The amelioration of neuroinflammation through mTOR-mediated immunometabolic reprogramming has great clinical significance in promoting disease recovery. HIF-1 is a key transcription factor in cells under hypoxia and plays a very important role in the adaptive response of cells to oxygen (77). Increased cellular uptake of glucose is induced by the overexpression of enzymes involved in glycolysis, and GLUT and mediates metabolic reprogramming.

##### 3.2.2.6 Other mechanisms, including lactylation

Although glycolytic reprogramming plays an important role in microglial polarization, whether this process affects microglial polarization has not yet been elucidated. In 2021, Luo and Wang et al. reported a close temporal and spatial correlation between surgical trauma-induced injury-associated microglial polarization and metabolic reprogramming in aged mice. Reprogramming of glucose metabolism is an important process in the regulation of microglial polarization and neuroinflammation (78). In 2022, Jiang and Wei et al. identified zinc finger E-box binding homeobox 1 (Zeb1) as a key regulator of glycolytic gene expression. Zeb1 promotes the transcription of enzyme-encoding genes through the PI3K/Akt/HIF-1α pathway under hypoxic conditions. In 2023, Zhai et al. identified NADPH oxidase 4 (NOX4) as a regulator of microglial metabolic reprogramming that promotes injury-associated microglial polarization by facilitating glycolytic processes through ROS production (79). As research continues to progress, the focus on the reprogramming of glucose metabolism has shifted to the glucose metabolite lactate. As an energy source and metabolic byproduct, lactate has unknown nonmetabolic functions in health and disease. In 2019, Zhang and Tang et al. reported that lactylation drives the expression of M2 genes during M1 macrophage polarization (80). Thus, glycolysis drives microglial injury-associated polarization, and proinflammatory factors that induce polarization also influence glycolysis. These findings suggest that immunity and metabolism are interrelated.

## 4 Microglial glycolytic reprogramming and neurological disorders

The abnormal activation of microglia leads to the loss or alteration of their normal functions. A growing body of research has demonstrated that microglia undergo polarization (**Table 1**) and glucose metabolic reprogramming upon stimulation in neuroinflammatory and neurodegenerative diseases. Targeting glycolytic pathways in disease contexts may therefore represent a novel therapeutic strategy. This article highlights representative studies on microglial glycolysis in neuroinflammatory and neurodegenerative diseases, as well as potential therapeutic strategies.

**Table 1:** Stimulation of microglial polarization in neurological diseases.

Diseases	Molecules	Mechanisms	References
Ischemic stroke	HMGB-1	Activating TLR-4 signaling exacerbates brain I/R injury and is a major activator of microglia after ischemic stroke	(145)
	Prx family proteins	Prx-1 is expressed in microglia and reduces microglial activation; Prx-5 reduces ROS levels and NFκB and MAPK activation in microglia	(146)
	Gal3	Activating microglia through the activation of TLR-4 amplifies the inflammatory response	(147)
	Hsp70	Intracellularly reduces pro-inflammatory factor signaling; extracellular binding to TLRs activates microglia	(83)
	ATP	The expression of the ATP receptors P2X4 and P2X7 is increased in microglia in a cellular stroke model; P2Y6 is activated by UDP and limits postinjury inflammation; the P2Y12 receptor is pro-inflammatory and chemotactic	(83)
AD	Aging	Activating the M1 phenotype leads to increased destructiveness in subjects with traumatic brain injury	(148)
	A-β	Aggregated Aβ activates M1 microglia via receptor for advanced glycation endproducts (RAGE) and TLRs; the induction of NLRP3 inflammasome activation contributes to M1 microglial activation	(149, 150)
	Tau protein	Stimulates M1 microglia polarization through NLRP3, which mediates IL-1β release	(151)
PD	α-syn	α-Syn released from degenerated DAergic neurons is an endogenous DAMP that activates microglia, and misfolded α-syn triggers a reactive M1-like pro-inflammatory phenotype	(152, 153)
	Human neuromelanin	Dual activation of NF-κB via the classical and p38MAPK transactivation pathways induces M1 formation	(154, 155)
ALS	Gal3	The induction of anti-inflammatory responses induces the microglial M2 phenotype and limits neuroinflammation and disease progression	(156)
	mSOD1	Stimulates M1 activation by CD14 and TLR	(157)
Neuropathic pain	MAPK signaling pathway	The phosphorylation of P38 MAPK stimulates M1 polarization	(158)
	DKK3	DKK3 blocks SNI-induced M1 microglial polarization and promotes the M2 phenotype	(159)
	ATP	The ATP receptors P2X4, P2X7, and P2Y12 are associated with neuropathic pain, and these receptors activate microglia by engaging different signals that converge to p38 MAPK	(160)

A-β, amyloid β-protein; α-syn, α-synuclein; DKK3, Dickkopf3; Gal3, galectin-3; HMGB-1, high mobility group box-1 protein; Hsp70, heat shock protein 70; MAPK, mitogen-activated protein kinase; mSOD1, mutant superoxide dismutase 1; Prx family proteins, peroxidase family proteins; SNI, spinal nerve injury.

### 4.1 Ischemic stroke

Stroke is the second leading cause of death and disability worldwide, and ischemic stroke is the most common type of stroke. Microglia play a crucial role in the development of ischemic stroke. They are activated in the acute stage of the disease and have complex effects on the poststroke phase. Ischemic brain injury activates microglia by increasing the production of ATP, heat shock protein 60, and glutamate (81–83). Microglia exhibit a dynamic response to ischemic brain injury. Microglia in ischemic stroke exhibit marked heterogeneity across different brain regions and at different stages of the disease (84). STING is predominantly activated in microglia following ischemic stroke. Inhibition of STING inhibits microglial polarization toward the injury-associated phenotype and attenuates cerebral ischemia–reperfusion-induced neuroinflammation and brain damage; it also reduces the release of mtDNA and prevents further expansion of brain damage after ischemia–reperfusion (85).

Modulating microglial polarization requires a large amount of energy, and proinflammatory microglial polarization activates glycolysis and the PPP. An experimental study revealed that microglia from the damaged brains of mice with permanent middle cerebral artery occlusion expressed genes related to glycolysis for 72 hours (86). Chemokine-like factor 1 (CKLF1) is a secreted protein. CKLF1 expression increases after cerebral ischemia (87). The number of activated microglia after acute stroke is reduced upon the knockdown or inhibition of CKLF1. Cell-based experiments have shown that CKLF1 increases microglial glycolysis and significantly decreases the level of OXPHOS and the maximal OXPHOS capacity of microglia. CKLF1 activation inhibits the phosphorylation of AMPK, increases mTOR phosphorylation, and induces the production of IFN-1α, suggesting that inflammatory responses activated by CKLF1 require AMPK/mTOR/HIF-1α pathway activation to promote glycolysis (88). HK has an irreplaceable role in glycolytic metabolism, and selective inhibition of HK2 blocks neuroinflammation caused by microglial activation, preventing ischemic brain injury and significantly reducing the infarct size 24 hours after the onset of ischemic stroke. Thus, HK2 may be a promising therapeutic target for the treatment of ischemic stroke-related neurological damage (62). Resolvin D1 (RvD1), a lipid mediator, has been shown to increase microglial numbers after ischemic stroke, enhance OXPHOS while suppressing glycolysis, and thereby provide sufficient energy to support microglial phagocytosis of neutrophils (89). 2-Deoxy-D-glucose (2-DG) is a glucose analog that can be phosphorylated by hexokinase, thereby competitively inhibiting the conversion of glucose to glucose-6-phosphate and suppressing glycolysis. The clinical safety of 2-DG has been thoroughly investigated (90). 2-DG has the ability to cross the blood–brain barrier (BBB), enabling it to effectively reach lesion sites and exert its therapeutic effects. *In vivo* experiments have demonstrated that treatment with 2-DG improves motor function in mice subjected to middle cerebral artery occlusion (MCAO) (91). In a randomized controlled trial involving non-diabetic patients with AD, metformin was found to improve learning and memory abilities (92). This effect may be associated with metformin-mediated activation of AMPK, which influences the TCA cycle; improving microglial metabolism may represent a potential area of interest (93).

### 4.2 Parkinson’s disease

PD is a chronic neurodegenerative disease characterized by α-synuclein (α-syn) aggregation and the death of dopaminergic neurons. However, shifts in the microglial phenotype are strongly influenced by neuroinflammation and PD progression. Early in PD, repair-associated microglia produce mainly anti-inflammatory factors that reduce inflammation and promote tissue repair. As the disease progresses, α-syn is released extracellularly by dopaminergic neurons. The activation of NADPH oxidase, proinflammatory factors, and ROS induces oxidative stress, which promotes the oxidation, aggregation, and propagation of α-syn in adjacent neurons, shifting microglia toward the injury-associated phenotype, causing persistent neurotoxicity and exacerbating disease progression (94, 95). Microglia can take up more glucose than other cells in the brain. Glucose uptake and microglial activity are positively correlated (96). Clinical evidence has shown disturbances in cerebral glucose metabolism in PD patients (97). α-Syn promotes glycolysis via pyruvate kinase repair-associated and inhibits OXPHOS, which contributes to the reprogramming of glycolysis in microglia. Preformed fibrils (PFFs) are preformed α-syn fibers, and stimulating microglia with PFFs can recapitulate PD. Acute exposure to PFFs results in injury-associated microglial polarization, increased expression of enzymes related to glucose uptake and glycolysis, and an elevated lactate content, which may be associated with the reprogramming of microglial metabolism from OXPHOS to aerobic glycolysis via the Akt/mTOR/HIF-1α pathway (98).

Methyl-4-phenyl-1,2,3,6-tetrahydropyridine is a neurotoxic compound commonly used to create animal models of PD. The possible mechanism could be the inhibition of mitochondrial complex 1 activity. The mitochondrial hydroxylase Clk1 plays an important role in electron transport and antioxidant activity in the mitochondrial respiratory chain. An experimental study suggested that Clk1 deficiency-induced increases in glycolysis and inflammatory responses are mediated by the mTOR/HIF-1α pathway. Although Ckl1 deficiency does not increase neuronal sensitivity to methyl-4-phenyl-1,2,3,6-tetrahydropyridine, Ckl1 deficiency contributes to the progression of PD by exacerbating microglia-mediated inflammation, which promotes dopaminergic neuron death. Therefore, a potential target for treating neuroinflammation in PD may be the regulation of Clk1 activity (99). Melatonin receptor 1 (MT1) is widely expressed in substantia nigra neurons and glial cells. Studies have found that activation of MT1 can significantly suppress LPS-induced neuroinflammation and reverse the excessive glycolysis and OXPHOS observed in microglia under LPS stimulation. Currently, clinically used MT1 agonists such as agomelatine, ramelteon, and tasimelteon are primarily prescribed for the treatment of sleep disorders. The development of MT1-specific agonists, however, may hold great potential for managing neurodegenerative diseases accompanied by sleep disturbances (100). Studies have shown that 2-DG participates in anti-neuroinflammatory responses via the AMPK–mTOR–IKK signaling pathway and alleviates dopaminergic (DA) neuronal loss in LPS- and MPTP-induced mouse models by suppressing neuroinflammation (56). In a model of postoperative neurocognitive disorder (PND) in aged mice, administration of 2-deoxy-D-glucose (2-DG) suppressed glucose metabolism reprogramming, thereby reducing surgery-induced increases in activated microglia and pro-inflammatory cytokines, ultimately leading to significant improvement in cognitive impairment (78).

### 4.3 Alzheimer’s disease

AD is a neurodegenerative disease. It is characterized by the formation of intracellular amyloid β (Aβ) aggregates and extracellular neurofibrillary tangles composed of hyperphosphorylated tau, leading to neuronal loss and memory impairment. In individuals with AD, activated microglia accumulate around damaged brain tissue, and microglia maintain the homeostasis of the internal environment of the brain by phagocytosing and removing Aβ around neurons and fragments of damaged neurons.

**Figure 3:**
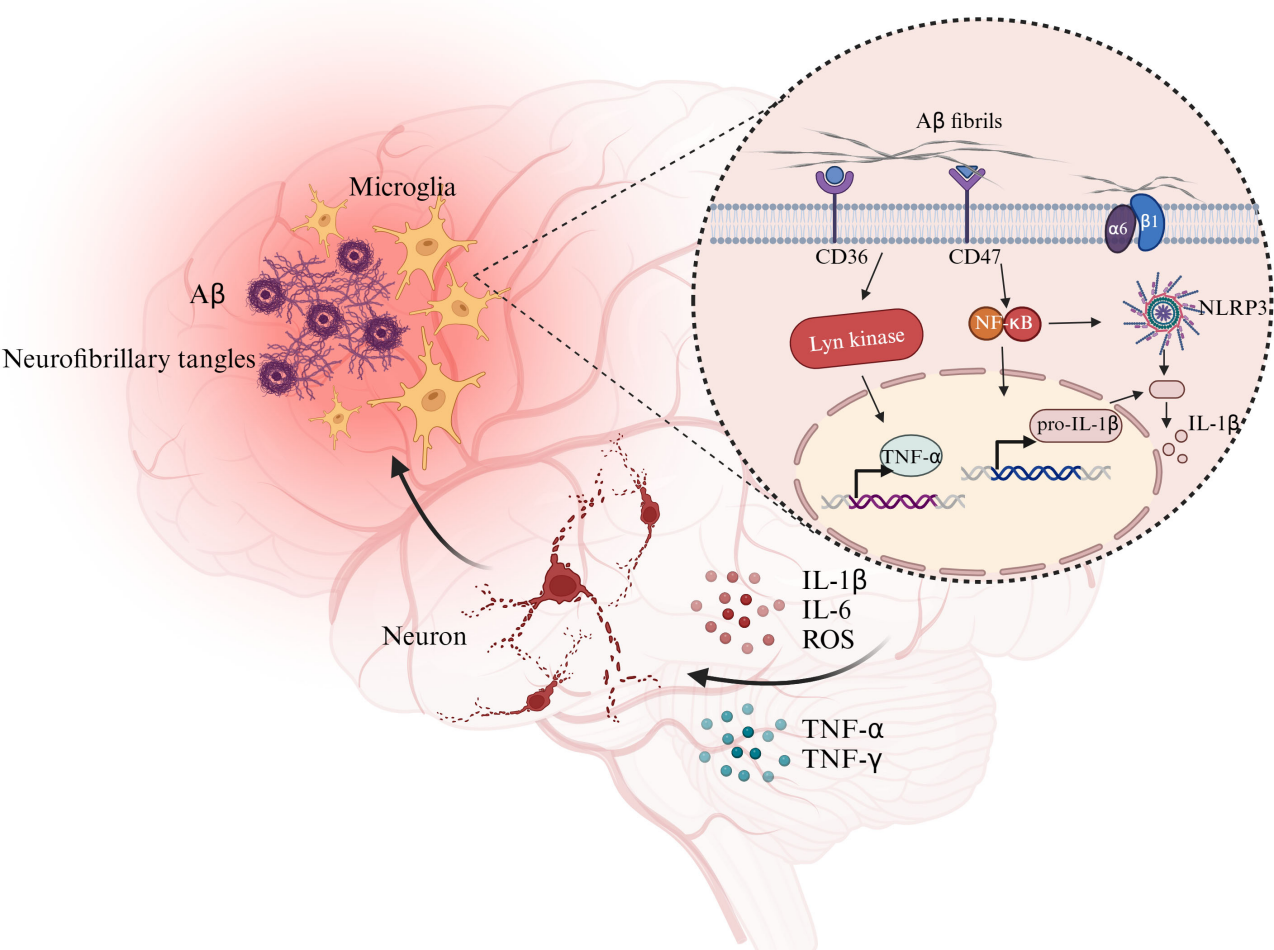
Glycolytic reprogramming and damage-related polarization processes in microglia. Resting microglia can be stimulated by PAMPs and DAMPs, undergo metabolic reprogramming (upregulated GLUT1, reduced oxidative phosphorylation, enhanced glycolysis and pentose phosphate pathway). Phagocytosed dsDNA activates the cGAS–STING pathway, with NF-κB involvement, leading to cytokine and interferon production and promoting microglial polarization and inflammatory responses. Abbreviation: cGAMP: cyclic GMP-AMP; cGAS: cyclic GMP-AMP synthase; ER, endoplasmic reticulum; F-1,6-2P, fructose-1,6-bisphosphate; GLUT1, glucose transporter protein 1; G-6-P, glucose-6-phosphate; IFN, interferon; IRF3, interferon regulatory factor 3; LDH, lactate dehydrogenase; NF-κB, nuclear factor kappa-light-chain-enhancer of activated B cells; OXPHOS, oxidative phosphorylation; PPP, pentose phosphate pathway; ROS, reactive oxygen species; TBK1, TANK-binding kinase1. Created with BioRender.com

The phagocytic activity of microglia requires cytoskeletal remodeling, which demands substantial energy (101). To meet the energetic demands of Aβ clearance, microglia—particularly those surrounding Aβ plaques—undergo a metabolic shift from OXPHOS to glycolysis (102) (**Figure 3**). As tau proteins continue to accumulate within neurons, affected neurons secrete neurotoxic cytokines to induce the proinflammatory polarization of microglia. Aβ also triggers acute inflammation in microglia by activating the NLRP3 inflammasome (2, 103, 104). Microglia are polarized toward the injury-associated phenotype, and the intracellular levels of the glycolytic markers HK, glucose-6-phosphate dehydrogenase, and phosphofructokinase 1 increase significantly. Significant decreases in the maximal mitochondrial respiratory capacity and mitochondrial fission indicate a shift from OXPHOS to glycolysis in microglia (40, 105). However, in early AD, the cytokines IL-4 and IL-10, glucocorticoids, and immune complexes polarize microglia toward the repair-associated phenotype. Microglia prevent AD by removing Aβ deposits through phagocytosis and by secreting insulin-degrading enzymes and other enzymes that degrade Aβ (106). These findings indicate the dual roles of microglia in AD.

A study has shown that cognitive impairment in AD patients is associated with abnormal glucose utilization, glycolysis, and OXPHOS in the brain (107, 108). The two most prominent metabolism-related features of AD are altered expression of glucose transporter proteins and insulin resistance (109, 110). Alterations in glucose metabolism usually occur early in the disease and can promote Aβ accumulation and tau protein phosphorylation. AD models have been used to test the effects of treatments targeting glucose metabolism. Liraglutide is an analog of glucagon-like peptide-1 and can enhance glucose transport (111). Semaglutide is a novel glucagon-like peptide-1 (GLP-1) receptor agonist. *In vitro* studies have demonstrated that semaglutide enhances glucose metabolism and promotes glycolysis, exerting its effects by regulating neuronal GLUT4 expression and activating the glucose–SIRT1 signaling pathway (112). Mullein increases the expression of GLUT3 and GLUT4 in microglia and promotes glucose transport (113). Dimethyl malonate (DMM), an inhibitor of succinate dehydrogenase (SDH), suppresses the expression of IL-1β and TNF-α by reducing glycolysis and inhibiting mitochondrial respiration in microglia (114). These findings suggest that restoring glucose metabolism may represent a potential therapeutic strategy for Alzheimer’s disease (AD) (115).

mTOR-dependent metabolic reprogramming is essential for glycolysis. Metabolic reprogramming via the Akt/mTOR/HIF/1α pathway impairs microglial energy metabolism, mitochondrial autophagy, and phagocytosis, resulting in deficits in dopaminergic neuron function. Metabolic reprogramming underlies the reactivity of microglia. The modulation of metabolism may be an effective strategy for altering microglial function and may also be a promising approach for treating AD. Despite the lack of AD-related studies, mTOR inhibitors are promising treatments for AD because they modulate microglial polarization (116). IFN-γ is a potent regulator of the mTOR pathway and glycolysis (117) and can cross the blood-brain barrier (118). Studies have shown that sodium rutin can enhance mitochondrial OXPHOS, providing microglia with sufficient energy to support the phagocytosis required for Aβ clearance. Moreover, it can effectively restore the suppressed OXPHOS function in pro-inflammatory microglia, thereby mitigating microglial glucose metabolic dysfunction under pathological conditions (119).

### 4.4 Multiple sclerosis

MS is an immune-mediated neurodegenerative disease characterized primarily by axonal demyelination in the central nervous system, accompanied by pronounced inflammatory immune responses and neurodegeneration within the lesion sites (93, 120, 121). In MS, the homeostatic microglial markers transmembrane protein 119 (TMEM119) and purinergic receptor P2Y G-protein–coupled 12 (P2RY12) decline sharply as the disease progresses (122). This indicates that microglia undergo state transitions during disease progression. The number and characteristics of microglia within lesions are often used to distinguish stages of MS progression. In active MS, the entire lesion area is densely populated with microglia, whereas in inactive or mixed MS, microglia are mainly distributed along the lesion borders (123). For a long time, MS treatment has primarily focused on the early-stage disruption of the blood–brain barrier (BBB), which allows peripheral B cells, T cells, and monocytes to infiltrate the CNS parenchyma. This infiltration triggers peripheral immune cell–mediated attacks on myelin within the central nervous system, ultimately leading to axonal demyelination (121). In acute MS lesions, prominent iron deposition is observed at the margins of microglia. The accumulated iron exerts pro-inflammatory effects, which may be related to increased intracellular ROS levels and subsequent alterations in mitochondrial morphology and function (124, 125). At the lesion margins, microglia predominantly rely on glycolysis as their main energy metabolism. Emerging research highlights another aspect of MS pathogenesis—namely, energy metabolism alterations in disease-associated cells. Transcriptomic analyses have suggested that iron accumulation in microglia can promote intracellular glycolytic activity (126, 127). This enables microglia to generate more ATP and drives their morphological alterations (123). Transcriptomic data indicate that the expression of glycolysis-related genes is increased in microglia within the gray matter of MS patients (127). A clinical study demonstrated that in MS patients, cerebrospinal fluid lactate levels are positively correlated with the number of MS plaques, showing a stronger association than with general CNS inflammatory markers. This finding suggests that inflammatory plaques may be closely linked to the processes of lactate production and release (128).

At present, there are relatively few drugs targeting glycolytic pathways for the treatment of MS, with dimethyl fumarate (DMF) being the most representative. DMF can be hydrolyzed by esterases in the small intestine into monomethyl fumarate (MMF), which is thought to exert antioxidant and anti-inflammatory effects by interfering with the TCA cycle. Although its precise mechanism remains unclear, treatment with DMF has been shown to significantly increase succinate levels. Among these metabolites, L-carnitine and acylcarnitines have been demonstrated to activate the Nrf2-mediated antioxidant pathway (129). In addition, both *in vivo* and *in vitro* studies have demonstrated that DMF can catalyze the succination of cysteine residues on the glycolytic enzyme GAPDH, leading to its inactivation. In other words, DMF downregulates glycolysis within cells and promotes a metabolic shift toward oxidative phosphorylation (130).

### 4.5 Traumatic brain injury

TBI refers to diffuse damage and neurodegeneration of the central nervous system caused by external mechanical forces. Its pathological features include mild multifocal axonal injury, microglial activation, and microhemorrhages. Microglia can become markedly activated within one week following trauma. Experiments in spinal cord injury models have shown that microglia at the injury site exhibit both injury-associated and repair-associated activation characteristics (84). In fact, microglia in the brain after TBI display heterogeneous characteristics, encompassing both reparative and injury-associated phenotypes. Under pathological conditions, microglia alter their metabolic state by redirecting glycolytic intermediates toward the pentose phosphate pathway (PPP), thereby generating NADPH and producing ROS via NOX (131). Fluorodeoxyglucose (FDG) positron emission tomography (PET) has demonstrated that significant alterations in cerebral glucose metabolism occur following TBI (132). In the early stages of the disease, cerebral glucose metabolism is characterized by heightened glycolysis, which subsequently shifts toward a reduced cerebral metabolic rate of glucose in the later stages (133). Experiments have shown that bromovalerylurea (BU) can reduce the expression of pro-inflammatory genes in microglia both *in vivo* and *in vitro*, while significantly inhibiting microglial glycolytic activity (134). This suggests that microglial glucose metabolism may play an important role in TBI-associated inflammation; however, current research on this topic remains limited.

### 4.6 Other central nervous system diseases

ALS is a neurodegenerative disease that slowly destroys motor neurons in the spinal cord, brainstem, and primary motor cortex, ultimately resulting in paralysis and death. Approximately 10% of ALS cases are familial, and superoxide dismutase 1 (SOD1) is the most common gene mutated in patients with familial ALS. Microglia play dual roles in ALS, exhibiting an repair-associated phenotype and protecting motor neurons during disease onset, and then transforming to the injury-associated phenotype and exacerbating motor neuron damage in the terminal phase. The intracellular and extracellular accumulation of misfolded mSOD1 may be a key factor in the conversion of microglia from the repair-associated phenotype to the injury-associated phenotype (1, 94, 135). PET findings suggest that widespread hypoglycemia is not associated with brain atrophy or aging in several brain regions of the motor cortex in ALS patients (136, 137). Several possible mechanisms underlying this phenomenon have been proposed, including reduced cerebral blood flow and defective glucose transport mechanisms or hexokinase activity. Data from experiments using the TAR DNA-binding protein (TDP-43)-overexpressing Drosophila model of ALS revealed increases in glycolysis and PPP activity (138). Reductions in lactate marker levels, lactate production and release, and PPP flux in SOD1 and TDP-43 expressing neurons indicate reduced glycolysis (139). Glycolysis defects are observed in the CNS tissues of symptomatic mutant SOD1G93A mice, but studies of changes in glucose metabolism in microglia are lacking (140). A model of Drosophila overexpressing TDP-43 revealed that the activation of glycolysis via GLUT3 results in neuroprotection and improved locomotion (138), suggesting that the glycolytic pathway may play a beneficial role in ALS; however, further research is needed to confirm this finding.

Somatosensory damage causes neuropathic pain; its pathogenesis is complex, involving entire neural pathways and all glial cell types, and it is characterized by nociceptive hypersensitivity and abnormal pain processing due to synaptic remodeling. Many studies have emphasized the relevance of the neuroimmune response in neuropathic pain. Chronic nerve injury allows microglia to be polarized toward the injury-associated phenotype. As inflammation increases, intracellular signaling pathways are activated and pain signaling is disrupted, resulting in systemic inflammation. The levels of inflammatory factors and microglial markers are reduced in repair-associated microglia, resulting in an uncontrolled inflammatory state (141). Various changes, such as the upregulation of proinflammatory factors, the generation of advanced glycosylation end products, and the activation of the mTOR/HIF/1α pathway, induce glycolysis in microglia (142, 143). Glycolytic reprogramming is critical for pain sensitization. Neuropathic pain is characterized by impaired OXPHOS and enhanced mitochondrial glycolysis due to glycolytic reprogramming. These changes induce phenotypic shifts in cells, contributing to increased neuroinflammation and oxidative stress in the periphery and CNS. Changes in the NAD/NADH ratio induced by the reprogramming of microglial glucose metabolism alter the redox state, decreasing the ratio of activated N-methyl-D-aspartic acid receptors and increasing Ca^2+^ influx, thereby triggering a downstream inflammatory cascade response (142, 144).

## 5 Conclusions

Microglia represent the first line of immune defense in the CNS, characterized by high activity and unique sensitivity (4). Under normal conditions, microglia maintain their own cellular homeostasis and perform housekeeping functions, continuously monitoring changes in the brain environment. Their unique sensitivity enables them to detect subtle alterations within the brain and mount active responses (6). With the advancement of technologies such as single-cell sequencing, the classification of microglia has gradually shifted from a simple dichotomy to a multidimensional framework based on specific environmental contexts. Such refined classification approaches facilitate a deeper understanding of microglial phenotypes and functions (14, 23, 24, 25, 161).

Metabolic alterations that occur in immune cells in response to immune stimulation are referred to as immunometabolism. As key immune cells in the CNS, microglia also undergo corresponding metabolic changes during the execution of their functions, among which glucose metabolic reprogramming—directly linked to energy supply—plays a pivotal role. On the one hand, glycolysis meets the high energy demands of different microglial phenotypes; on the other hand, its metabolic byproduct lactate exerts effects through post-translational modification in the form of lactylation, showing temporal dynamics—promoting pro-inflammatory gene expression in the early phase while suppressing inflammation-related genes and exhibiting reparative properties in the later phase (44, 46). During microglial phenotypic transitions accompanied by glycolysis, glycolysis can influence these changes through multiple pathways—including GLUT (59, 60), MCT (46, 64)., HK2 (61), mitochondria (69), the PI3K signaling pathway (38, 76), and lactylation (14, 23–25, 161)—thereby enabling microglia to better adapt to diverse pathological stimuli.

Extensive research has been conducted on microglial immunometabolism in both acute and chronic neurological diseases; however, current studies on microglia still face certain limitations. At present, most studies on the pathological states of microglia rely on LPS-stimulated BV-2 cells. In fact, studies have already indicated substantial differences between primary microglia and the BV-2 cell line in response to strong LPS stimulation, with primary cells exhibiting more robust and complex responses compared to BV-2 cells (162). In reality, stimulation with LPS alone can hardly recapitulate the complex pathological context of disease states. The regulatory mechanisms by which metabolic intermediates influence disease progression and microglial phenotypic transitions remain unclear. Strategies for reprogramming microglial glucose metabolism at different stages of neurological diseases remain to be further explored. In addition, based on the hypothesis of mitochondrial compartmentalization, it remains to be clarified whether microglia also exhibit functional specialization across different spatial and temporal contexts, stages, and regions in neurological diseases. In addition to elucidating the underlying mechanisms, drug delivery remains a critical challenge due to the unique characteristics of the BBB. Currently, the integration of nanomaterials with pharmacological approaches offers the potential to enhance drug penetration across the BBB and achieve targeted delivery to pathological sites (163, 164). Although no drugs are currently available in clinical practice that specifically target microglial glucose metabolism for the treatment of neurodegenerative diseases, ongoing research may eventually establish this approach as a novel therapeutic direction.
